# Efficacy and safety of lenvatinib plus pembrolizumab in patients with advanced and recurrent endometrial cancer: a systematic review and meta-analysis

**DOI:** 10.3389/fimmu.2024.1404669

**Published:** 2024-08-09

**Authors:** Guangwei Yan, Yanmin Du, Huanhuan Zhang, Jinxiang Yan, Yixuan Liu, Zhenying Ban, Yong-Zhen Guo, Xianxu Zeng

**Affiliations:** ^1^ Department of Pathology, The Third Affiliated Hospital of Zhengzhou University, Zhengzhou, China; ^2^ Zhengzhou Key Laboratory of Early Diagnosis for Gynecological Diseases, Zhengzhou, China

**Keywords:** lenvatinib, pembrolizumab, immunotherapy, endometrial cancer, meta-analysis

## Abstract

**Background:**

Various trials have demonstrated the clinical benefits of lenvatinib plus pembrolizumab in patients with advanced or recurrent endometrial cancer, regardless of mismatch repair (MMR) status or histologic subtype. The majority of the previously published trials had small sample sizes. Here, we aimed to assess the reported efficacy and safety profile of lenvatinib plus pembrolizumab in patients with advanced and recurrent endometrial cancer.

**Methods:**

We utilized the Cochrane Library, PubMed, Web of Science and Embase databases to identify clinical trials evaluating the efficacy and safety of lenvatinib plus pembrolizumab in patients with advanced and recurrent endometrial cancer. The outcomes analyzed were progression-free survival (PFS), overall survival (OS), the objective response rate (ORR), the disease control rate (DCR) and the incidence of adverse events (AEs). Subgroup analysis was conducted on the basis of MMR status (deficient, dMMR or proficient, pMMR).

**Results:**

Four trials (582 patients) were included. The pooled ORR was 32.7% [95% confidence interval (CI): 28.9–36.5]. Subgroup analysis revealed an ORR of 48.1% (95% CI: 26.1–70.2) for dMMR group and 33.1% (95% CI: 25.7–40.6) for pMMR group. The pooled DCR was 74.9% (95% CI: 71.3–78.4%). Subgroup analysis revealed a DCR of 81.0% (95% CI: 64.5–97.6) for the dMMR group and 76.3% (95% CI: 66.3–86.3) for the pMMR group. Follow-up was reported in all included studies. The median range time of PFS and OS was 5.3 months-258 days and 17.2 months-not reached, respectively. Regarding safety, the overall pooled proportions of any-grade AE and AEs ≥ grade 3 were 95.8% (95% CI: 89.5–100.0) and 80.2% (95% CI: 59.9–100.0), respectively.

**Conclusion:**

Lenvatinib plus pembrolizumab showed a relevant clinical benefit and significant toxicity in patients with advanced and recurrent endometrial cancer. Further studies encompassing long-term outcomes are warranted.

**Systematic review registration:**

https://www.crd.york.ac.uk/PROSPERO/display_record.php?RecordID=522160/, identifier CRD42024522160.

## 1 Introduction

Endometrial cancer (EC) is the sixth most common malignancy in women, with continually increasing incidence and disease-related mortality rates (1). The prognosis for women who present with advanced-stage or multifocal recurrent disease is poor because of a lack of major treatment advances (2). Currently available therapeutic options for advanced and recurrent EC are palliative. EC is considered a heterogeneous malignancy with diverse histologic, clinical, and molecular features according to The Cancer Genome Atlas (TCGA) (3). No standard therapy for advanced or recurrent EC after the failure of standard first-line chemotherapy has been globally accepted (4). Molecular characterization has advanced our understanding of the role of different immunotherapeutic strategies (5, 6). In particular, immune checkpoint inhibitors (ICIs, e.g., durvalumab and pembrolizumab) (7) have established efficacy in EC patients with deficient mismatch repair (dMMR). As mentioned above, up to 70% of EC patients present with proficient mismatch repair (pMMR) (3); therefore, assessing the role of immunotherapy in patients with pMMR is crucial.

Lenvatinib in combination with pembrolizumab is endorsed with Category 1 evidence by the National Cancer Care Network (8) and is a widely accepted and guideline-endorsed approach for treating EC patients with pMMR after the failure of platinum-based chemotherapy (9, 10). Although several clinical trials have either been completed or are ongoing, real-world evidence supporting the use of lenvatinib plus pembrolizumab in patients with advanced and recurrent EC remains limited. In addition, not all women can tolerate immunotherapy with lenvatinib plus pembrolizumab, as toxicities are common (11). At present, the majority of the available data have been generated from studies with small sample sizes based on heterogeneous populations. Therefore, we performed a systematic review and meta-analysis to investigate the potential benefits in terms of the objective response rate (ORR), disease control rate (DCR), and safety when utilizing lenvatinib plus pembrolizumab, thus providing a more stable and reliable reference for the application of lenvatinib plus pembrolizumab in the treatment of advanced and recurrent EC.

## 2 Materials and methods

### 2.1 Search strategy and selection criteria

The Embase, Cochrane Library, PubMed and Web of Science databases were searched from database inception until March 13, 2024. The search string was (“PD-1 inhibitor” OR “immunotherapy” OR “immune checkpoint inhibitor” OR “pembrolizumab”) AND (“lenvatinib” OR “lenvatinib mesylate” OR “lenvima”) AND (“endometrial cancer” OR “endometrial carcinoma” OR “endometrium cancer” OR “endometrial neoplasms”). In addition, we searched the references of related articles to find other relevant publications. Two independent authors (GWY and YMD) screened the titles and abstracts of the identified studies. The protocol was registered in the International Prospective Register of Systematic Reviews (number: CRD42024522160).

The criteria for inclusion in this meta-analysis were as follows: (1) studies including patients with advanced or recurrent EC; (2) studies in which the treatment regimen was lenvatinib plus pembrolizumab; (3) prospective or retrospective clinical studies (including randomized controlled trials and single-arm trials); and (4) studies with sufficient data for quantitative analysis, such as data on progression-free survival (PFS), overall survival (OS), the ORR, the DCR, and the incidence of adverse events (AEs). The exclusion criteria were as follows: (1) review papers, case reports, and preclinical experiments; (2) studies with overlapping or repeated data; (3) studies with research data that could not be extracted; (4) clinical trials without formally published articles; and (5) studies that were not published in English.

### 2.2 Data extraction and quality of evidence

The following data were extracted from each eligible study: the first author, publication year, number of participants, median follow-up, MMR status, ORR, DCR, median PFS, median OS, and incidence of AEs (any-grade AEs, AEs ≥grade 3). The quality of each trial was assessed using two well-established tools: theCochrane risk-of-bias tool for randomized trials (RoB 2) (12) and the risk of bias in non-randomized studies of interventions (ROBINS-I) tool (13).

### 2.3 Statistical analysis

Statistical analysis was performed via R software version 4.2.3 (“meta” package). Statistical heterogeneity was assessed via the *I* ^2^ and Cochran Q chi-square tests. If high heterogeneity existed (*I* ^2^ >50% or *p <*0.1), a random effects model (REM) was used; otherwise, a fixed effects model (FEM) was used (14). We calculated the ORR, DCR and AEs using the combined ratio method, with pooled effect size measure and a 95% CI. Subgroup analysis was carried out according to MMR status. Sensitivity analysis was performed to assess the influence of two retrospective studies on the meta-analysis results. A *p* value <0.05 was considered statistically significant.

## 3 Results

### 3.1 Study selection and characteristics

**Figure 1:**
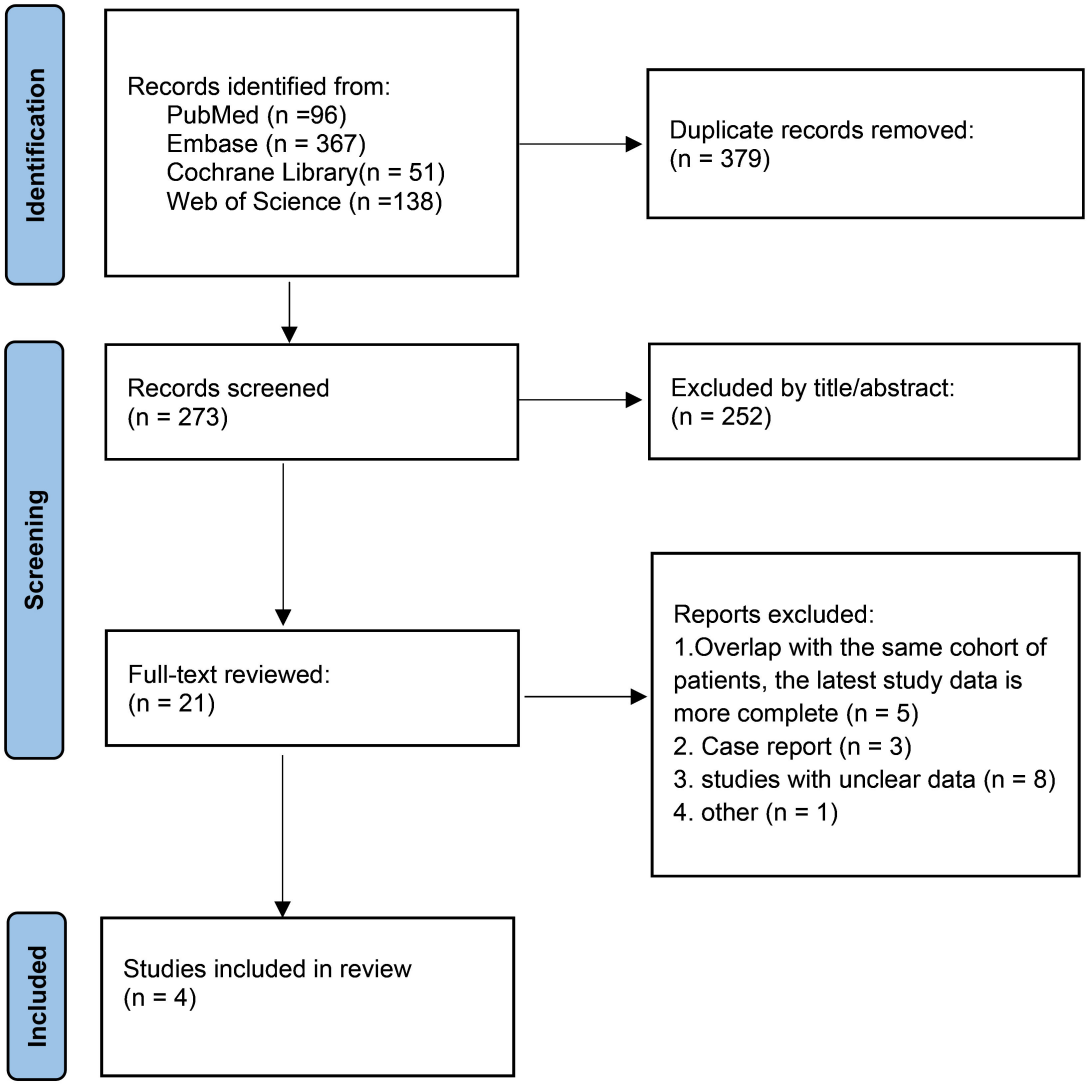
PRISMA flow diagram of the selection process.

A PRISMA study flow diagram is presented in **Figure 1**. A total of 652 relevant publications were identified via the search strategy, which was reduced to 273 after the removal of duplicates. After title and abstract review, 21 potentially relevant studies were eligible for full-text review. Ultimately, a total of 4 clinical trials involving 582 patients were included in the meta-analysis (9, 10, 15, 16). The methodological assessment of the included studies is presented in **Supplementary Table 1**. Overall, low and moderate risk of bias were achieved for all included articles.

All included studies were published between 2022 and 2023. One study was a phase-III clinical trial of lenvatinib plus pembrolizumab compared with physicians’ choices of chemotherapy (10), and the other three studies were single-arm trials (9, 15, 16). The sample size varied greatly among the included trials, ranging from 15 to 411 participants. For lenvatinib plus pembrolizumab treatment, the initial recommended dose was 200 mg pembrolizumab intravenously every 3 weeks and 20 mg lenvatinib once daily in all included studies. The studies and variables of interest are summarized in **Table 1**.

**Table 1:** Characteristics of the included studies in the meta-analysis.

Study	Year	Sample size	Age (year, median)	MMR status			Objective Response Rate (n, %)			Disease Contral Rate (n, %)			Median PFS (month, median)			Median OS (month, median)			Follow-up time (month, median)
				dMMR	pMMR	unknown	overall	dMMR	pMMR	overall	dMMR	pMMR	overall	dMMR	pMMR	overall	dMMR	pMMR	
KEYNOTE-146 (9)	2023	108	65.1	11	94	3	43/108	7/11	36/94	89/108	10/11	77/94	7.4	26.4	7.4	17.7	NE	17.2	34.7
Tochigi M (15)	2023	15	66	1	8	6	6/15	—	—	11/15	—	—	8.6	—	—	—	—	—	11.3
KEYNOTE-775 (10)	2023	411	64	65	346	0	131/411	26/65	105/346	296/411	48/65	248/346	7.3	10.7	6.7	18.7	31.9	18	18.7
Kim J (16)	2022	48^a^	62.5	2	44	2	10/42	—	—	32/42	—	—	5.3	—	—	NR	—	—	6.9

a,44 patients available for the analysis; NE, not estimable; NR, not reached.

### 3.2 Objective response rate

**Figure 2:**
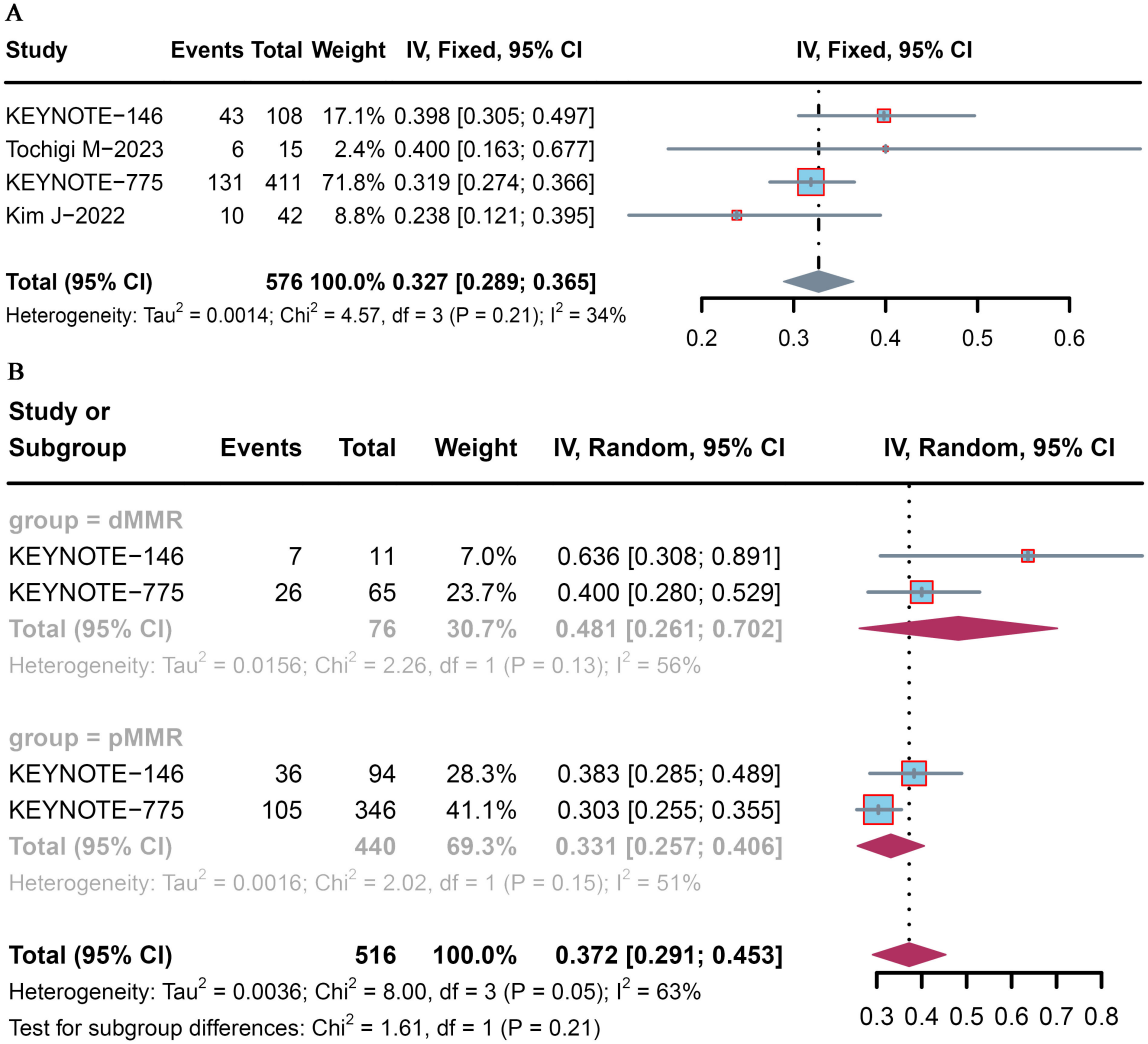
Pooled ORR for pembrolizumab plus lenvatinib immunotherapy in patients with advanced or recurrent EC. Forest plot of the **(A)** overall population and **(B)** dMMR and pMMR populations.

All four trials analyzed the ORR, which ranged from 23.8% to 40.0%. The pooled ORR of patients with advanced or recurrent EC who received lenvatinib plus pembrolizumab was 32.7% (95% CI: 28.9–36.5) (**Figure 2A**). Among the included studies, that by Kim et al. (16) reported the lowest ORR (23.8%), whereas that by Tochigi et al. (15) reported the highest ORR (40.0%).

Subgroup analysis was conducted according to MMR status. The pooled proportions of the ORR were 48.1% (95% CI: 26.1–70.2) and 33.1% (95% CI: 25.7–40.6) for advanced and recurrent EC patients with dMMR and pMMR, respectively (**Figure 2B**).

### 3.3 Disease control rate

**Figure 3:**
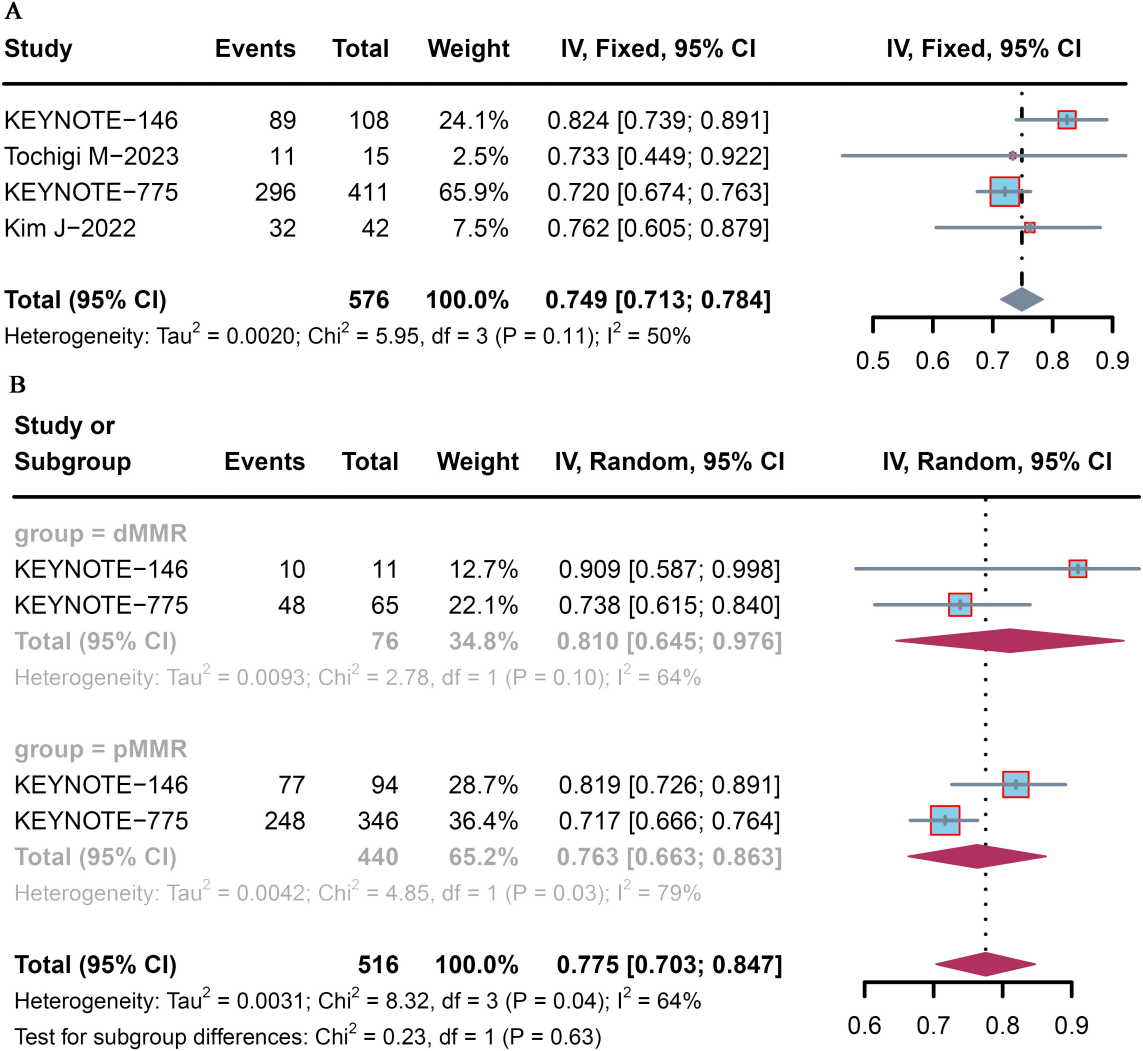
Forest plot of the DCR for pembrolizumab plus lenvatinib immunotherapy in patients with advanced or recurrent EC. Forest plot of the **(A)** overall population and **(B)** dMMR and pMMR populations.

All four trials analyzed the DCR, which ranged from 72.0% to 82.4%. The pooled DCR of patient with advanced or recurrent EC who received lenvatinib plus pembrolizumab was 74.9% (95% CI: 71.3–78.4) (**Figure 3A**). Among the included studies, that by KEYNOTE-146 (9) reported the highest DCR (82.4%), whereas that by KEYNOTE-775 (10) reported the lowest DCR (72.0%).

Subgroup analysis was conducted according to MMR status. The pooled DCR was 81.0% (95% CI: 64.5–97.6) and 76.3% (95% CI: 66.3–86.3) for advanced and recurrent EC patients with dMMR and pMMR, respectively (**Figure 3B**).

### 3.4 Progression-free survival and overall survival

Follow-up was conducted in all included studies, and the follow-up time ranged from 6.9 months to 34.7 months. All studies included in the analysis reported PFS after the administration of pembrolizumab plus lenvatinib immunotherapy, and the median PFS time ranged from 5.3 months to 258 days. With respect to OS, data were available in three studies, and the median OS time ranged from 17.2 months to not reached. Survival outcomes were assessed based on MMR status in two included studies (9, 10). In the dMMR subgroup, the median range time of PFS and OS was 10.7–26.4 and 31.9-not reached, respectively. In the pMMR subgroup, the median range time of PFS and OS was 6.7–7.4 and 17.2–18.0, respectively.

### 3.5 Adverse effects

**Figure 4:**
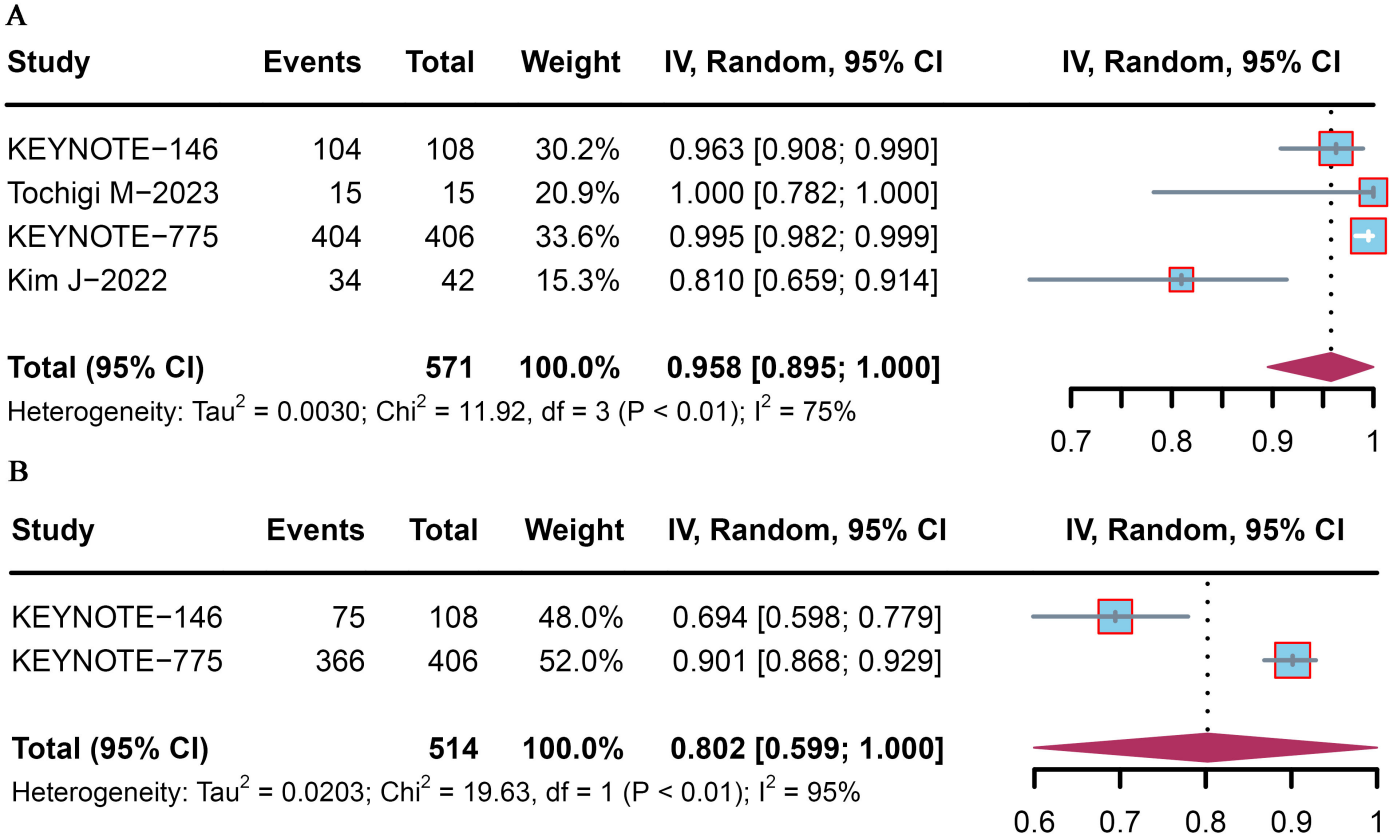
Pooled incidences of adverse events associated with pembrolizumab plus lenvatinib immunotherapy in patients with advanced or recurrent EC. Forest plot of **(A)** the overall incidence of adverse events and **(B)** the incidence of adverse events≥ grade 3.

Safety and toxicity were assessed via any-grade AEs in four trials and AEs ≥ grade 3 in two trials (**Table 2**, **Supplementary Table 2**). The overall pooled incidences of any-grade AEs and AEs ≥ grade 3 were 95.8% (95% CI: 89.5–100.0) and 80.2% (95% CI: 59.9–100.0), respectively (**Figures 4A, B**).

**Table 2:** Any grade and grade ≥ 3 adverse events reported in the included studies in the meta-analysis.

Study	KEYNOTE-146 (9) (n=108)		Tochigi M (15) (n=15)		KEYNOTE-775 (10) (n=406)		Kim J (16) (n=48)	Overall^a^ (n=577)	
	ang grade	grade 3/4	ang grade	grade 3/4	ang grade	grade 3/4	ang grade	ang grade	grade 3/4
Overall	105	75	15	—	405	366	34	559	441
Hypertension	66	35	12	6	264	159	8	350	200
Hypothyroidism	48	1	14	0	239	6	7	308	7
Diarrhea	57	7	4	0	226	33	—	287	40
Nausea	43	3	—	—	210	14	—	253	17
Decreased appetite	51	0	—	—	189	31	—	240	31
Vomiting	29	0	—	—	153	12	—	182	12
Weight decreased	28	2	—	—	144	44	—	172	46
Fatigue	56	9	8	4	138	22	9	211	35
Arthralgia	34	2	—	—	131	7	2	167	9
Proteinuria	24	4	5	4	124	21		153	29
Constipation	—	—	—	—	115	3	—	115	3
Anemia	14	6	—	—	114	28	—	128	34
Urinary tract infection	—	—	—	—	112	17	—	112	17
Headache	22	0	—	—	107	2	—	129	2
Thrombocytopenia	—	—	10	2	—	—	—	10	2
Liver dysfunction	—	—	9	4	—	—	—	9	4
Loss of appetite, anorexia	—	—	6	4	—	—	—	6	4
Fever	—	—	6	0	—	—	—	6	0
Hand-foot syndrome	—	—	6	2	—	—	—	6	2
Renal impairment	—	—	4	0	—	—	—	4	0
Electrolyte imbalance	—	—	3	2	—	—	—	3	2
Skin symptoms	29	5	3	0	—	—	4	36	5
Stomatitis	36	0	—	—	—	—	—	36	0
Dysphonia	30	0	—	—	—	—	—	30	0
Vomiting	29	0	—	—	—	—	—	29	0

a Only AEs occurring in >25% of patients are included in the table.

AEs of interest occurred in 96.9% of all 577 patients, with the following frequencies: hypertension (60.66%), hypothyroidism (53.38%), diarrhea (28.76%), nausea (49.74%), and decreased appetite (43.85%). The most common AE ≥ grade 3 was hypertension (34.66%), followed by decreased weight (7.97%), diarrhea (6.93%), fatigue (6.07%), and decreased appetite (5.37%).

### 3.6 Sensitivity analysis

Because two retrospective studies including 15 patients and 40 patients were included, which might have impaired the quality of the meta-analysis, we performed a sensitivity analysis to assess the influence of the two retrospective trials on the meta-analysis results. After the retrospective trials were omitted, the pooled results did not significantly differ (**Supplementary Figure 1**), indicating that the results were stable.

## 4 Discussion

Advances in the understanding of the molecular classification of EC have recently paved the way for immunotherapeutic strategies (17). Several completed or ongoing trials assessing the safety and effectiveness of single-agent targeted therapies or therapies combining multiple medications for managing patients with advanced or recurrent EC with dMMR and pMMR have been conducted (9–11, 18–20). Given the emerging evidence that antiangiogenic agents modulate the tumor microenvironment (TME) and synergize with ICIs (21, 22), the combination of PD-1/PD-L1 inhibitors and antiangiogenic therapy is an attractive treatment strategy for EC patients with pMMR (5). In the KEYNOTE-146/Study 111, treatment with lenvatinib (an oral tyrosine kinase inhibitor) plus pembrolizumab had compelling antitumor activity in patients with advanced EC, irrespective of microsatellite instability (MSI) status (9). These findings support the accelerated approval of lenvatinib plus pembrolizumab as a new second-line treatment for EC patients with pMMR by the Food and Drug Administration (FDA).

In this meta-analysis involving 4 clinical trials and 582 patients, we evaluated the efficacy and safety of lenvatinib plus pembrolizumab in patients with advanced and recurrent EC via data from published studies. In early-phase clinical trials of cancer immunotherapy, intermediate endpoints, such as the ORR and DCR, have been routinely used as surrogates for outcomes (23–25). The results of this meta-analysis revealed that the pooled ORR was 32.7%. Subgroup analysis revealed an ORR of 33.1% for the difficult-to-treat pMMR group. These results indicated that the combination of lenvatinib plus pembrolizumab has a higher ORR than other reported immunotherapies in similar populations (18, 19, 26). The pooled DCR was 74.9%, and subgroup analysis revealed a DCR of 81.0% for the dMMR group and 76.3% for the pMMR group. As such, the favorable ORR and DCR suggest that lenvatinib plus pembrolizumab has clinically meaningful improvements in this patient population, irrespective of MMR status.

The most common AE of any grade was hypertension (60.66%), followed by hypothyroidism (53.38%), diarrhea (28.76%), nausea (49.74%), and decreased appetite (43.85%). These results were generally similar to those reported in the global study population (9, 10). In another meta-analysis involving EC patients who received PD-1/PD-L1 inhibitor monotherapy (24), the most common treatment-related AE of any grade was fatigue (19.77%), followed by nausea (13.33%), diarrhea (13.10%), anemia (11.95%), and hypothyroidism (9.77%). When considering only AEs ≥ grade 3, patients receiving lenvatinib plus pembrolizumab had a greater incidence of hypertension (34.66%), followed by decreased weight (7.97%), diarrhea (6.93%), fatigue (6.07%), and decreased appetite (5.37%). Patients who received PD-1/PD-L1 inhibitor monotherapy had a higher incidence of anemia (1.84%), diarrhea (1.84%), and hyperglycemia (0.92%) (24). Notably, Tochigi et al. reported that thrombocytopenia occurred in 66.6% (n=15) of patients and that the incidence decreased to 20.0% with a dose reduction (15); however, large-scale studies are needed to support this result, as it was not estimable in the other three included studies. Although the frequency of AEs was greater in the group that received lenvatinib plus pembrolizumab immunotherapy, the toxicity profile was manageable with supportive care and medications, modifications or interruptions (27, 28). The clinical team should follow successful AE management strategies to increase tolerance and quality of life for patients while maximizing potential therapeutic benefits (11, 27, 28).

Overall, our meta-analysis confirmed the well-established role of lenvatinib plus pembrolizumab for the treatment of previously treated, recurrent, and advanced EC regardless of MSI status. The ongoing phase-III ENGOT-en9/LEAP-001 study is evaluating the combination of lenvatinib plus pembrolizumab versus paclitaxel and carboplatin in the first-line setting in patients with advanced or recurrent EC. This global study includes sites in the USA, Austria, China, Canada, and Germany, and positive results will demonstrate a similar clear benefit of pembrolizumab in combination with lenvatinib in terms of PFS and OS in both the pMMR and all-cancer populations (29). ICIs are moving from second-line and beyond to first-line treatment regimens. The incorporation of anti-PD1 or anti-PD-L1 agents into platinum-based chemotherapy for advanced and metastatic EC substantially improves oncologic outcomes, especially within the MMRd/MSI-H subset (30, 31). Given the recent approvals of pembrolizumab and dostarlimab in combination with chemotherapy as first-line treatments for recurrent/metastatic EC, lenvatinib plus pembrolizumab and chemoimmunotherapy with initial treatment are more beneficial for survival in the pMMR and dMMR populations, respectively. However, further research is needed to explore these aspects.

This meta-analysis has several limitations. First, given that three of the four included studies had a single-arm design, they have a high risk of selection bias and performance bias. Second, the subgroup analysis on the basis of MMR status included data from only two of the four included studies. Third, data on long-term clinical outcomes, such as the 5-year OS and PFS rates, were not available in the included studies, likely due to the relatively short duration of follow-up. Nevertheless, an updated analysis encompassing OS and PFS data is imperative.

## 5 Conclusion

This meta-analysis of 4 clinical trials involving 582 patients with advanced or recurrent EC confirmed the therapeutic benefit of lenvatinib plus pembrolizumab in terms of the ORR and DCR, irrespective of MMR status. The results of the current meta-analysis highlighted the reliable efficacy of lenvatinib plus pembrolizumab in advanced or recurrent endometrial cancer, thus supporting its broad clinical application. Further analysis from updated trials is needed to clarify the impact of immunotherapy on OS and PFS.

## Data Availability

The original contributions presented in the study are included in the article/**Supplementary Material**. Further inquiries can be directed to the corresponding author.

## Supplementary material

Supplementary Figure 1 Pooled estimates for the overall population excluding the two retrospective trials.
