# Supplementary figures and images for: Efficacy and safety of lenvatinib plus pembrolizumab in patients with advanced and recurrent endometrial cancer: a systematic review and meta-analysis
Source: Front Immunol. 2024 Aug 9;15:1404669. doi: 10.3389/fimmu.2024.1404669 (PMC11341375; doi:10.3389/fimmu.2024.1404669)

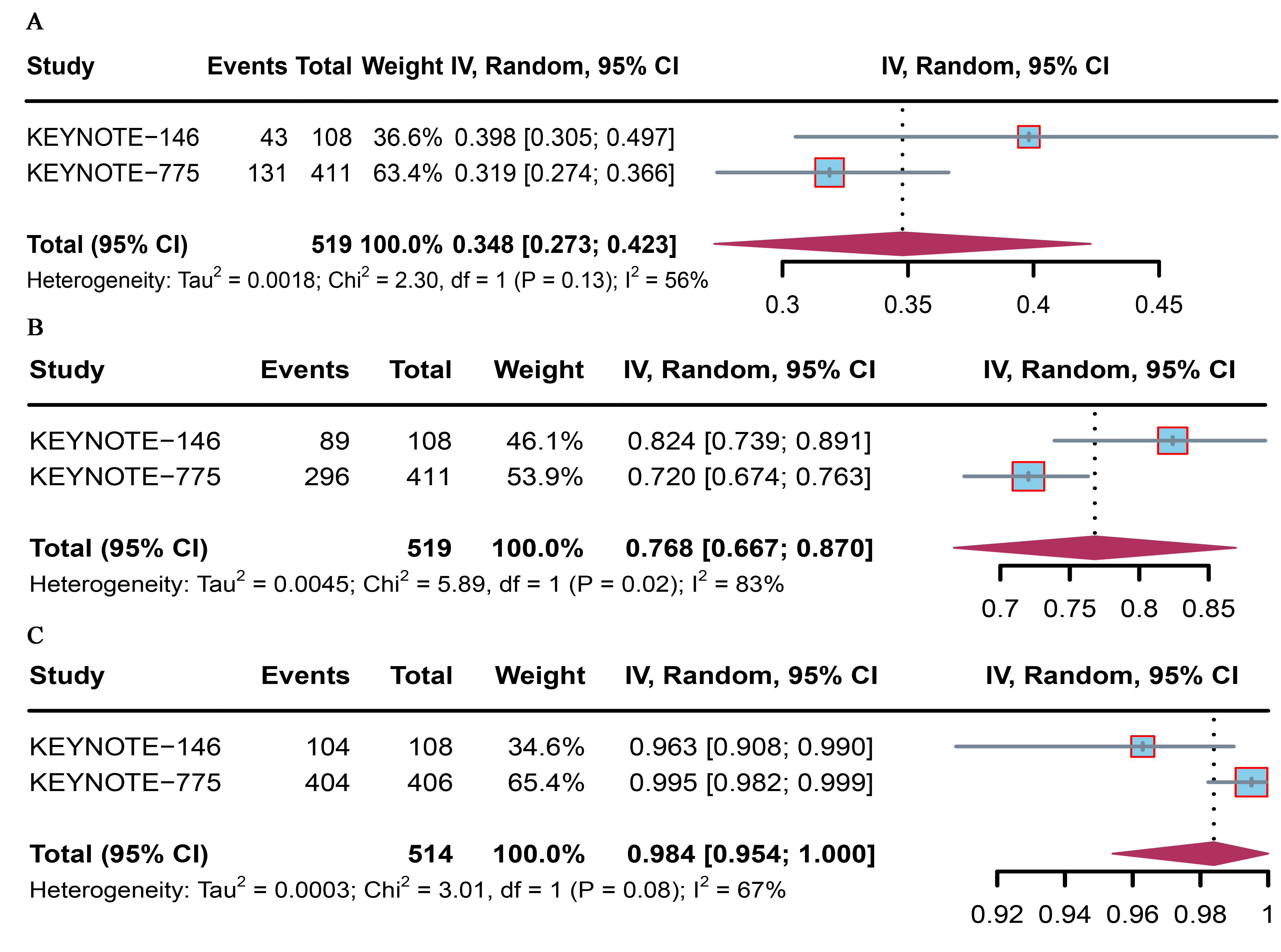

Supplement: Supplementary Figure 1 — Pooled estimates for the overall population excluding the two retrospective trials. [file Image_1.tif]
